# Tomato secondary metabolites as natural regulators of *Bemisia tabaci* behavior and performance: current applicability and prospects

**DOI:** 10.3389/fpls.2025.1704832

**Published:** 2026-01-15

**Authors:** Victor Hugo Maldonado Machado da Cruz, Thiago Rutz da Silva, Paulo Gimenez Cremonez, André Luiz Biscaia Ribeiro da Silva, Jesui Vergilio Visentainer, Camila Rodrigues

**Affiliations:** 1 Department of Horticulture, Auburn University, Auburn, AL, United States; 2 Department of Chemistry, State University of Maringá, Maringá, Paraná, Brazil; 3 Department of Entomology and Plant Pathology, Auburn University, Auburn, AL, United States

**Keywords:** acylsugars, integrated pest management, secondary metabolites, *Solanum lycopersicum*, terpenes, whitefly

## Abstract

The sweetpotato whitefly (*Bemisia tabaci*) is one of the most damaging insect pests of tomato (*Solanum lycopersicum*), often causing severe yield losses and transmitting viral pathogens such as Tomato Yellow Leaf Curl Virus. Although chemical insecticides are commonly used for whitefly management, the rapid development of insecticide resistance in *B. tabaci* poses a major challenge to sustainable control. In contrast, wild tomato genotypes exhibit superior resistance, primarily attributed to the production of secondary metabolites (SMs), such as terpenoids, phenolic compounds, and acylsugars. These natural compounds affect whitefly behavior, feeding, development, and survival through both deterrent (antixenosis) and toxic or growth-inhibiting (antibiosis) mechanisms. This review investigates the roles of terpenes, phenolic compounds, nitrogen-containing SMs, and acylsugars in tomato resistance to *B. tabaci*, with a particular emphasis on their antixenosis and antibiosis effects. It also highlights recent advances in the characterization and application of these compounds to support sustainable whitefly management and guide resistance breeding strategies. A better understanding of the synergistic effects among metabolite classes and their integration with other control strategies could enable the development of tomato genotypes with enhanced and durable resistance to *B. tabaci*.

## 1 Introduction

Tomato (*Solanum lycopersicum* L.), an herbaceous plant native to western South America, is cultivated globally to meet consistent, year-round consumer demand (Caruso et al., 2022; Kumaraswamy et al., 2024). Despite its widespread production, tomato remains highly susceptible to several insect pests that significantly threaten both yield and quality (Vosman et al., 2018). Among these pests, the sweetpotato whitefly (*Bemisia tabaci* Genn.; Hemiptera: Aleyrodidae) is particularly destructive, with severe infestations capable of reducing yields by up to 90% (Pal et al., 2021; Rahman Shah et al., 2023). Its impact is exemplified by reported economic losses exceeding $125 million in Florida’s tomato production and yield reductions of up to 35% in Georgia’s squash crops in 2016 (Abubakar et al., 2022).

The standard strategy for managing whitefly populations in tomato cultivation is the application of synthetic insecticides (Perring et al., 2018; Santegoets et al., 2021; Abubakar et al., 2022). However, the extensive use of these chemicals has led to the development of resistance in *B. tabaci* populations, particularly to insecticides such as pyriproxyfen and neonicotinoids, thereby reducing the long-term effectiveness of chemical control (Dempsey et al., 2017). To prevent or delay pest resistance development, chemical control should be combined with non-chemical approaches, including biological control, crop rotation, and host-plant resistance, as a part of an integrated pest management (IPM) strategy (Dempsey et al., 2017; Horowitz et al., 2020; Pal et al., 2021).

Wild relatives of *S. lycopersicum* possess a range of natural defense mechanisms that deter whitefly feeding or impair insect development (Wink, 2003, Wink, 2018). However, modern breeding programs have primarily focused on enhancing yield and fruit quality, often neglecting important morphological and biochemical characteristics associated with insect resistance. As a result, many defensive traits have been lost in cultivated varieties (Huang et al., 2022; Wang et al., 2024b). Reintroducing these resistance traits into commercial tomato germplasm could offer an effective strategy for managing *B. tabaci* infestations (Vosman et al., 2018; Santegoets et al., 2021; Kumaraswamy et al., 2024).

Several studies attribute the superior resistance observed in wild tomato genotypes to a higher density of glandular trichomes, which are specialized epidermal structures that function both as mechanical barriers and as reservoirs for secondary metabolites (SMs) (Zhang et al., 2020; Pal et al., 2021; Santegoets et al., 2021; Almeida et al., 2023; Rahman Shah et al., 2023; Rutz et al., 2024). These metabolites include terpenoids, phenolic compounds, acylsugars, and steroidal alkaloids, which contribute significantly to host plant resistance in the *Solanaceae* family (Zhang et al., 2020; Pal et al., 2021; Santegoets et al., 2021; Almeida et al., 2023; Rahman Shah et al., 2023; Rutz et al., 2024).

In recent years, numerous studies have focused on identifying SMs in various tomato genotypes that exhibit resistance to whiteflies. The goal is to enhance host plant resistance by utilizing these naturally occurring compounds as part of a sustainable pest management strategy. Therefore, understanding the roles of SMs in tomato–whitefly interactions is crucial for developing pest-resistant cultivars. This review synthesizes current knowledge on the major classes of SMs involved in tomato resistance to *B. tabaci*, with a focus on their antixenosis (deterrent) and antibiosis (toxic or growth-inhibiting) effects. It highlights recent advances in the characterization and practical application of these compounds to support sustainable whitefly management and inform future breeding efforts. By advancing the understanding of tomato chemical defenses, this review contributes to the development of whitefly-resistant cultivars and the implementation of more sustainable pest management strategies.

## 2 Sweetpotato whitefly (*B. tabaci*) and crop damage

The sweetpotato whitefly, *B. tabaci* (Gennadius) (Hemiptera: Aleyrodidae), is a polyphagous, sap-sucking insect that poses significant threats to global agriculture. It infests more than 1,000 plant species across at least 60 plant families, including economically important crops such as cassava, tomato, eggplant, cucurbits, cruciferous vegetables, okra, black pepper, sunflower, pulses, tobacco, groundnut, soybean, potato, cotton, lettuce, and various ornamentals (Navas-Castillo et al., 2011; Li et al., 2021; Abubakar et al., 2022; Perier et al., 2022);. While crops like corn are considered non-preferred hosts, *B. tabaci* can still complete its reproductive cycle on them, allowing year-round persistence and reinfestation on nearby susceptible plants (Quintela et al., 2016).

Taxonomically, *B. tabaci* is considered a cryptic species complex comprising over 40 morphologically indistinguishable species grouped into 11 major genetic groups (LIU et al., 2012; Sani et al., 2020; Brown et al., 2023). Among these, the New World whitefly (previously known as Biotype A), Middle East-Asia Minor 1 (MEAM1) whitefly (formerly Biotype B or *B. argentifolii* Bellows and Perring), and the Mediterranean whitefly (previously known as Biotype Q) are most relevant to tomato production (Perring et al., 2018). MEAM1, in particular, has spread globally since its introduction in the 1980s via ornamental plants, now establishing populations across Europe, the Mediterranean Basin, Africa, Asia, Central America, North America (Mexico and the USA), South America (Argentina, Brazil, Colombia, and Venezuela), and the Caribbean Basin (Perring, 2001).

*B. tabaci* reproduces rapidly, often producing multiple overlapping generations within a single growing season. Its reproductive strategy includes arrhenotokous parthenogenesis (arrhenotoky), where unfertilized females produce haploid males, allowing recessive resistance alleles to be immediately expressed (Byrne and Devonshire, 1996; Horowitz et al., 2003). This mechanism accelerates the spread of insecticide resistance and compromises novel technologies such as RNA interference (Shelby et al., 2020).

Crop damage caused by *B. tabaci* can be direct or indirect, both resulting in substantial economic losses (Sani et al., 2020). Direct damage occurs when nymphs and adults feed on plant sap, removing essential nutrients and reducing photosynthetic activity. This leads to symptoms such as chlorosis, premature leaf drop, and overall plant decline, particularly during heavy infestations (Gangwar and Gangwar, 2018). Additionally, nymphs secrete enzymes that interfere with plant physiology, causing irregular fruit ripening and inhibiting internal coloration. The honeydew secreted during feeding further exacerbates crop damage by promoting the growth of sooty mold on leaves and fruits, which reduces photosynthesis and degrades crop quality (Sani et al., 2020).

Indirectly, *B. tabaci* is an efficient vector of plant viruses (Jones, 2003). In tropical, subtropical, arid, and Mediterranean regions, the *Bemisia* and *Trialeurodes* genera are the major contributors to the transmission of 200 plant viruses, primarily those in the *Begomovirus* genus. These viruses can cause crop losses ranging from 20% to 100%, making whitefly-mediated transmission a major concern (Jones, 2003; Navas-Castillo et al., 2011). Among them, Tomato Yellow Leaf Curl Virus (TYLCV), transmitted by the *B. tabaci* MEAM1, is one of the most destructive viral pathogens of tomato (*S. lycopersicum*) in the southeastern U.S (Gautam et al., 2022). TYLCV infection leads to leaf yellowing and curling, stunted growth, and drastic reductions in fruit production and quality, with yield losses reaching 100% in severe outbreaks (Yan et al., 2021). Whiteflies also transmit other viruses, such as Tomato Chlorosis Virus (ToCV), a *Crinivirus* responsible for interveinal chlorosis, tissue necrosis, and reduced productivity (Lu et al., 2023).

Given the wide host range, high reproductive capacity, and efficient virus transmission, *B. tabaci* remains a persistent and complex challenge for crop protection. Effective management requires integrated strategies that combine vector suppression, the development and deployment of virus-resistant tomato cultivars, and the implementation of sustainable IPM practices to mitigate both direct damage and virus spread.

## 3 Current approaches to whitefly management

Chemical insecticides remain the primary strategy for managing *B. tabaci* populations in agricultural systems. These include chemicals from multiple Insecticide Resistance Action Committee (IRAC) classes, such as nerve and muscle-acting chemicals, e.g., neonicotinoids (IRAC 4A) and butenolides (IRAC 4D); anthranilic diamides, such as cyantraniliprole and chlorantraniliprole (IRAC 28); insect growth regulators, such as pyriproxyfen (IRAC 7C) and buprofezin (IRAC 16); and ketoenols, such as spiromesifen and spirotetramat (IRAC 23) (Perring et al., 2018; Abubakar et al., 2022; IRAC, 2024).

However, the widespread and repeated use of these insecticides has led to the development of resistance across nearly all insecticide classes, including organophosphates, carbamates, pyrethroids, neonicotinoids, diamides, and insect growth regulators (Bass et al., 2015; Horowitz et al., 2020). In major tomato-producing regions of the southeastern U.S., field populations of *B. tabaci* MEAM1 have shown localized resistance to spirotetramat and emerging resistance to afidopyropen (IRAC 9D) and cyantraniliprole (Dimase et al., 2024). Although several insecticides, including neonicotinoids (IRAC 4A), flupyradifurone (IRAC 4D), and cyantraniliprole, remain largely effective in Georgia (Cremonez et al., 2023), a reduction in imidacloprid (IRAC 4A) performance suggests the ongoing development of resistance in regional whitefly populations (Perier et al., 2024).

The limitations of chemical control have emphasized the need for IPM strategies that reduce pesticide dependence and promote sustainable practices. IPM approaches incorporate physical, mechanical, biological, and chemical methods to manage pest populations while minimizing environmental and non-target impacts (Angon et al., 2023).

Physical and mechanical strategies include the use of UV-reflective mulches and row covers to deter whiteflies and other vectoring pests, along with postharvest sanitation to eliminate residual populations (Qureshi et al., 2007; Simmons et al., 2010; Rutz et al., 2023). Reflective mulches affect whitefly behavior by altering their phototactic responses to light wavelengths (Fakhari et al., 2020), with silver plastic mulch demonstrating reductions in whitefly abundance and virus incidence, ultimately improving tomato yield and fruit quality (Csizinszky et al., 1999).

Biological control plays a vital role in IPM by leveraging natural enemies to regulate pest populations (Eilenberg et al., 2001; Zaviezo and Muñoz, 2023). Strategies include classical biological control (introducing exotic natural enemies), augmentative control (mass-rearing and periodic release of beneficial organisms), and conservative biological control (manipulating habitats to favor resident predators and parasitoids) (Le Hesran et al., 2019). These approaches reduce reliance on chemical inputs while promoting ecological stability (Jonsson et al., 2008; El-Wakeil et al., 2017).

Host plant resistance (HPR) is another essential component of IPM. Resistant cultivars carry genetically inherited traits that reduce herbivore damage and influence tritrophic interactions (Stout, 2014). HPR contributes to long-term pest suppression and plays a critical role in addressing global agricultural challenges related to climate change, population growth, and environmental degradation (Sharma and Ortiz, 2002; Mutschler et al., 2023). To effectively integrate HPR in IPM, breeding programs must first identify and screen germplasm for resistance traits. This includes evaluating cultivars, accessions, or lines that exhibit pest resistance or phenotypes associated with defense mechanisms (Sharma and Ortiz, 2002; Stout, 2014). The development of reliable screening protocols is vital to ensure the success of resistance breeding efforts.

## 4 Natural defense mechanisms of *Solanum lycopersicum* L.

Due to their sessile nature, plants have evolved a variety of defense mechanisms to protect themselves from herbivores and other environmental threats (Wink, 2003, Wink, 2018). Although the intrinsic defense mechanisms of plants vary based on morphology, population, and development stage, they can be summarized into morphological traits (e.g., barks, thorns, spikes, stinging hairs, and open growth) and biochemical mechanisms, involving the production and release of various allelochemicals, which are natural compounds that can deter or harm herbivores (Wink, 2018; War et al., 2020; Yactayo-Chang et al., 2020).

Recent studies have highlighted the significance of leaf structure in mediating *S. lycopersicum* resistance to *B. tabaci* (Zhang et al., 2020; Pal et al., 2021; Santegoets et al., 2021; Almeida et al., 2023; Rahman Shah et al., 2023). Traits such as leaf shape, thickness, and trichome density have been previously correlated with whitefly host preference. Tomato genotypes with narrower, thinner leaves and greater density of trichomes have been negatively correlated with *B. tabaci* populations (Pal et al., 2021). Conversely, increased leaf surface area and thickness have been positively correlated with higher populations of whitefly nymphs and adults, suggesting a preference for plant genotypes with these traits, likely due to increased palatability (Pal et al., 2021). The protective role of trichomes has been consistently highlighted as a key factor in whitefly resistance (Zhang et al., 2020; Pal et al., 2021; Santegoets et al., 2021; Almeida et al., 2023; Rahman Shah et al., 2023).

Trichomes are epidermal protrusions at the surface of plants that exhibit different sizes, shapes, and arrangements. These structures are broadly categorized into non-glandular and glandular types, which are further classified into eight subtypes (I-VIII) based on morphology (Li et al., 2023). Non-glandular trichomes primarily serve as physical barriers against whiteflies, hindering locomotion, feeding, and oviposition (War et al., 2012; Pal et al., 2021; Li et al., 2023). Glandular trichomes, however, provide dual functionality by serving as physical barriers and by producing and storing both inducible and constitutive SMs, which disrupt whitefly metabolism, reproduction, and behavior (War et al., 2020; Yactayo-Chang et al., 2020).

Comparative analyses between wild and cultivated tomato genotypes further confirm the significance of trichome-mediated defenses. Almeida et al. (2023) demonstrated that wild genotypes exhibited greater resistance to whiteflies due to higher densities of glandular trichomes, particularly type IV. Similar results were observed by Kumaraswamy et al. (2024), who observed greater antixenosis and antibiosis resistance in wild accessions than cultivated ones during free-choice and non-choice oviposition assays. Zhang et al. (2020) emphasized the contribution of type I, IV, and VI glandular trichomes to insect resistance, attributing their effectiveness to a combination of physical barriers and the secretion of deterrent compounds. In a broad assessment of tomato species, Zeist et al. (2021) identified a negative correlation between the type IV and VI glandular trichomes and whitefly populations in larval, nymphal, and adult phases.

The resistance conferred by glandular trichomes is attributed mainly to their capacity to produce a diverse range of SMs. These compounds can disrupt insect physiology and behavior, ultimately reducing infestation levels and crop damage (Wink, 2018; Afroz et al., 2021).

## 5 Secondary metabolites of tomato

Plant defense chemical mechanisms were initially categorized based on their response to microbial attack in phytoalexins and phytoanticipins. Phytoalexins were categorized as compounds synthesized in response to physical stress, while phytoanticipins were produced in anticipation of biotic stress (Yactayo-Chang et al., 2020). However, as research expanded beyond plant-microbe interactions, these endogenous plant defensive chemicals were more broadly classified based on their biosynthetic timing into inducible and constitutive SMs (Yactayo-Chang et al., 2020; Yang et al., 2023a).

Constitutive SMs are continuously synthesized and stored in tissues prone to herbivory, acting as an immediate response to biotic stress (Böttger et al., 2018). In contrast, inducible SMs are produced in response to specific biotic stress, such as insect saliva or oviposition fluids, detected by the plant during herbivore attack (Böttger et al., 2018; Afroz et al., 2021). Although the delay associated with inducible defenses may present a disadvantage, the chemical information collected by the plant in response to biotic stress allows for optimal adjustment to deal with the stress source (Kessler, 2015; Yactayo-Chang et al., 2020). In some cases, biosynthetic pathways are primarily constitutive, with inducibility restricted to the final step, as observed in the synthesis of cyanogenic glycosides (Afroz et al., 2021). Similarly, Riahi et al. (2023) demonstrated that herbivore-induced stress in early developmental stages can stimulate the enhanced production of glandular trichomes and acylsugars, thereby increasing host plant resistance.

**Figure 1:**
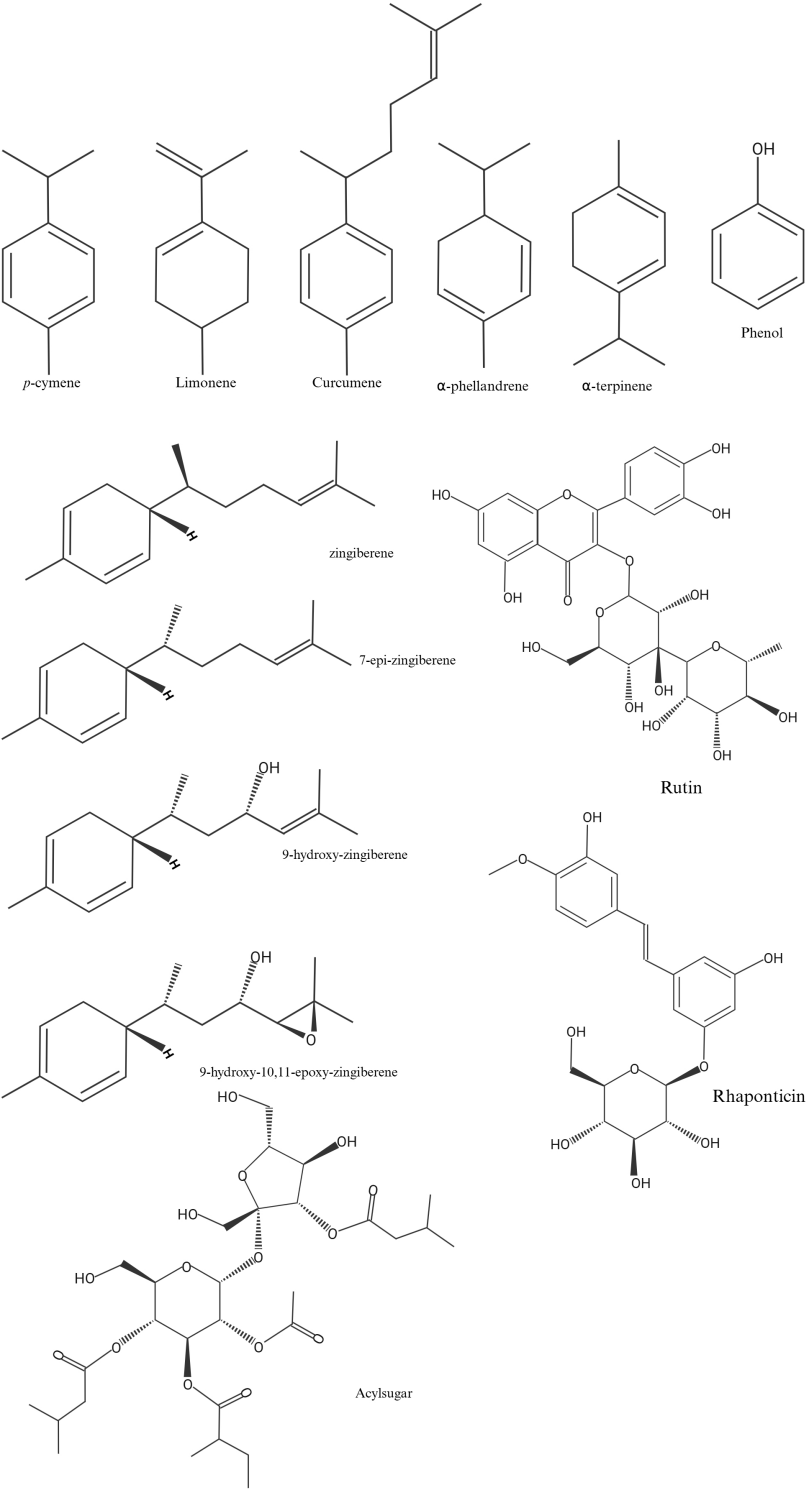
Chemical structures of secondary metabolites in *Solanum lycopersicum* (tomato) associated with resistance to *Bemisia tabaci* (whitefly). This figure was created with BioRender.com.

Plants produce an overwhelming diversity of SMs, which are fundamental to their ecophysiological function and adaptive responses to biotic and abiotic stresses (Moghe et al., 2023). This chemical diversity originates from a typical core structure submitted along the pathway to several derivatization steps, such as acylation, glycosylation, methylation, hydroxylation, and prenylation, among others (Wang et al., 2019). As a result, plant SMs are commonly categorized into three major groups: terpenes, phenolic compounds, and nitrogen-containing compounds (**Supplementary Table S1**; **Figure 1**) (War et al., 2020; Zhan et al., 2022; Razzaq et al., 2023). However, several key defense-related metabolites found in the *Solanaceae* family are not included in the abovementioned groups. Among them are acylsugars, a class of allelochemicals produced in glandular trichomes. These compounds exhibit broad-spectrum activity against arthropod pests and are associated with insect resistance across species in the *Solanum* genus, constituting a vital component of tomato’s natural chemical defense system (Dias et al., 2016; Fan et al., 2019; Moghe et al., 2023; Mutschler et al., 2023; Smeda et al., 2023).

### 5.1 Terpenes

**Figure 2:**
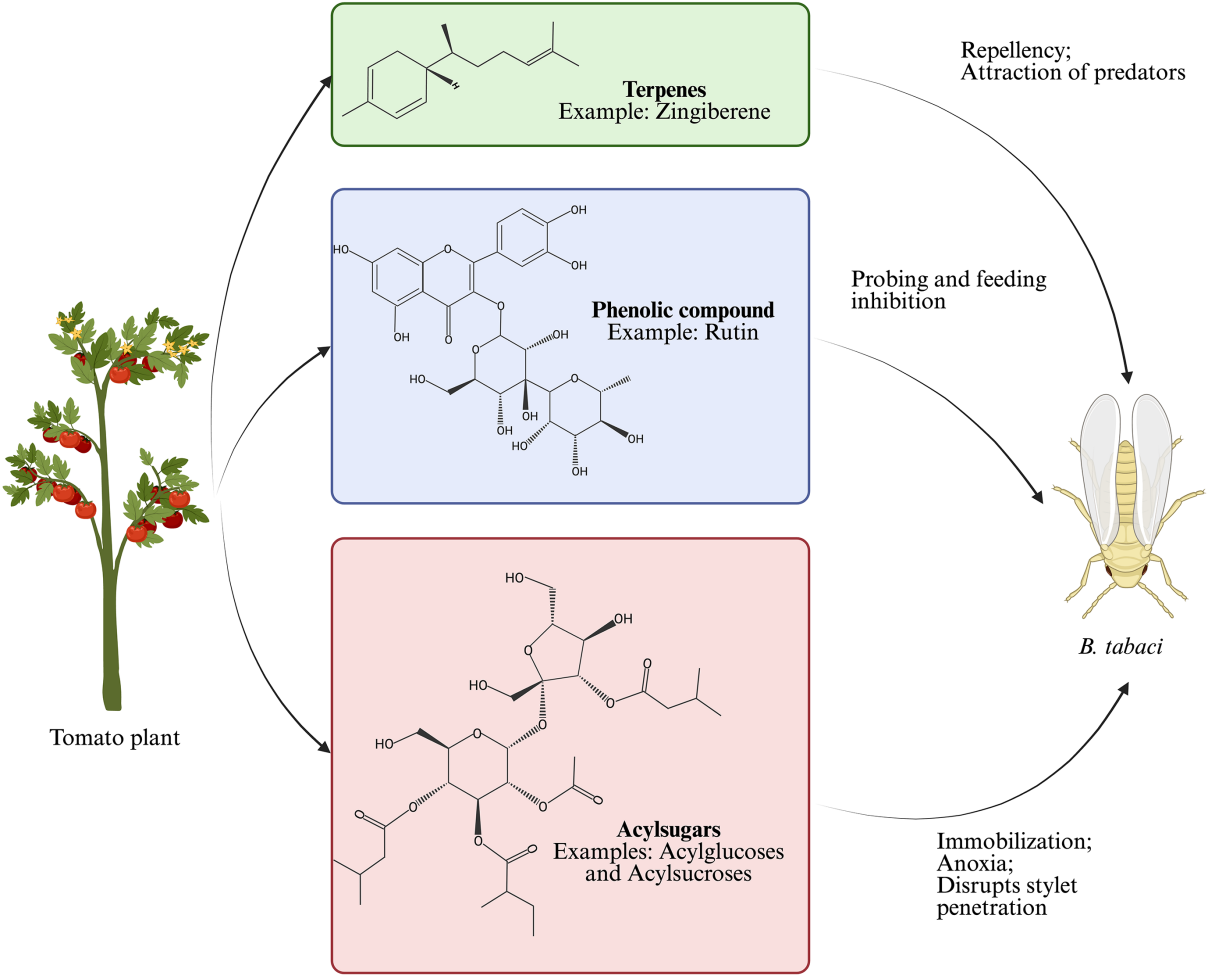
Schematic representation of key tomato secondary metabolites and their effects on the sweetpotato whitefly (*Bemisia tabaci*). This figure was created with.

Terpenes are a structurally diverse class of SM composed of polymeric hydrocarbons derived from isoprene units (Kabera et al., 2014; Razzaq et al., 2023). They are classified based on the number of isoprene units in their molecular backbone: monoterpenes (2 units), sesquiterpenes (3), diterpenes (4), triterpenes (5), tetraterpenes (8), and polyterpenes (more than 8) (War et al., 2020). These basic terpene backbones can be further derivatized upon the catalytic activity of enzymes, such as methyltransferases and acyltransferases, resulting in the formation of terpenoids. Terpenoids are structurally related to terpenes but contain additional functional groups, increasing their chemical diversity and biological activity (Pichersky and Raguso, 2018). Due to their similarity, the term “terpenoids” is often used in literature to refer to both terpenes and terpenoids. Several terpenoids have been associated with resistance to *B. tabaci* in tomato. Bleeker et al. (2009) investigated the role of plant volatiles and the interaction between whitefly and tomato cultivars, and identified sesquiterpenes such as zingiberene and curcumene as compounds that influence host preference. These volatiles act as repellents, deterring whiteflies from probing the plant (**Figure 2**). Similarly, monoterpenes such as *p*-cymene, α-terpinene, and α-phellandrene have been reported to exhibit repellent properties by altering insect behavior and reducing the attractiveness of certain tomato genotypes (**Supplementary Table S2**) (Bleeker et al., 2009, Bleeker et al., 2011; Conboy et al., 2019, Conboy et al., 2020).

In a subsequent study, Bleeker et al. (2011) demonstrated that specific stereoisomers, namely 7-epi-zingiberene and its dehydrogenated derivative R-curcumene, displayed significant repellent activity. These sesquiterpenes function as semiochemicals, signaling host toxicity. Under free-choice conditions, *B. tabaci* adults were less attracted to plants emitting these volatiles, indicating that the presence of 7-epi-zingiberene and R-curcumene alters insect decision-making.

Further characterization of resistant genotypes by Zabel et al. (2021) identified 7-epi-zingiberene derivatives, namely 7-epi-9-hydroxy-zingiberene and 7-epi-9-hydroxy-10,11-epoxy-zingiberene, in leaf exudates of tomato accession LA216. In no-choice bioassays, increasing concentrations of these sesquiterpenoids on tomato leaf discs correlated negatively with whitefly survival, confirming their bioactivity.

The use of terpenes and terpenoids in IPM has also been explored. Conboy et al. (2019) investigated intercropping tomato with marigolds, a non-host of whiteflies, which is rich in volatiles. In two large-scale glasshouse trials simulating commercial conditions, marigold companion planting initiated at the beginning of the growth period significantly reduced whitefly populations. However, introducing marigolds later as an emergency measure during heavy infestations had minimal effect. In a follow-up study, Conboy et al. (2020) investigated the substitution of synthetic pesticides with limonene, the primary volatile compound found in marigolds. Applications of limonene, alone or combined with methyl salicylate, improved crop yield under high pest pressure, with limonene alone resulting in a 32% yield increase.

Similarly, Xu et al. (2024) investigated the effects of varying concentrations of caryophyllene, an endogenous terpenoid of tomato, on the volatile organic compound profile and the resistance of tomato leaves to *B. tabaci*. The results demonstrated that caryophyllene facilitated the synthesis of volatile organic compounds, acting as a signaling factor and inducing plant defense mechanisms. Moreover, treatment with 25 μmol L^-1^ of caryophyllene over 12 h provided the most substantial resistance to whiteflies. There is also evidence in the literature of terpenes exuded due to biotic stress being assessed as a kairomone-based lure for natural predators of tomato pests (Ayelo et al., 2021).

Terpenoids influence whitefly behavior by manipulating environmental odor cues, which are crucial for host recognition (Bleeker et al., 2009). By emitting specific volatiles, plants can discourage insect settling and oviposition (Razzaq et al., 2023). Some monoterpenoids, such as iridoids and their derivates, can deter insect feeding by conferring a bitter taste to plants and denaturing protein molecules through imide formations, reducing nutritional value (Park et al., 2010; Afroz et al., 2021). Moreover, terpenoids exhibit neurotoxic effects on insects by targeting acetylcholinesterase (AChE), γ-Aminobutyric Acid (GABA), octopamine receptor, voltage-gated sodium channels, and glutamate-gated chlorine channels (Abdelgaleil et al., 2024).

### 5.2 Phenolic compounds

Phenolic compounds are a chemically varied and diverse group of SMs characterized by the presence of one or more phenol functional groups in their structure (Marchiosi et al., 2020). The term “phenolic compounds” encompasses chemicals synthesized from the shikimic/phenylpropanoid pathway, which provides several phenolic compounds, or the acetate/malonate pathway, responsible for producing simpler phenols (Cheynier et al., 2013).

Phenolic compounds can be classified based on the number of hydroxyl groups, chemical structure, and the nature of substitutions on their carbon skeletons (Kabera et al., 2014; Silva et al., 2023). Structurally, they range from simple monophenols to more complex oligophenols and polyphenols, such as benzoic acid, stilbenes, and tannins (Cheynier et al., 2013; Marchiosi et al., 2020) Based on substitution patterns, phenolics may consist of a simple benzene ring, an aromatic ring with an aliphatic chain, or two aromatic rings linked by a carbon chain (Hoang Anh et al., 2021). Polyphenols, which include both flavonoid and non-flavonoid classes, exhibit especially complex structures and are among the most widely distributed allelochemicals in plants (Kabera et al., 2014; Liu et al., 2023).

Among the major groups of SMs, phenolic compounds are widely distributed and ubiquitously present in all plant species, playing an active role in plant defense (War et al., 2020). These allelochemicals can display contact toxicity or be oxidized into toxic compounds, such as reactive oxygen species, which can affect insect growth and development (**Supplementary Table S2**) (Wink, 2003; Razzaq et al., 2023). Additionally, phenolic compounds, such as tannins, contain numerous hydroxyl groups that enable them to bind covalently to gut proteins in herbivorous insects, disrupting digestive processes and altering the gut microbiota (**Supplementary Table S2**) (Wink, 2003; Razzaq et al., 2023).

Studies have shown that phenolic compounds actively contribute to tomato resistance against *B. tabaci*. Anu et al. (2021) demonstrated that higher phenol and tannin concentrations in tomato plants were correlated with increased resistance to *B. tabaci*. These findings were supported by Pal et al. (2021), who screened tomato genotypes from eastern India and observed a negative correlation between *B. tabaci* population (nymph and adult) and both trichome density and total phenol content. In contrast, levels of proteins, reducing sugars, and total sugars were positively correlated with *B. tabaci* population build-up.

The functional significance of specific endogenous phenolic compounds has also been investigated. Su et al. (2018) demonstrated that *B. tabaci* infestation led to modifications in flavonoid composition of tomato plants, reducing concentrations of rutin, kaempferol rhamnoside, quercetin trisaccharide, and 3-O-methyl myricetin. These changes influenced subsequent host preference by conspecific whiteflies, suggesting these compounds contribute to induced resistance. A follow-up study by (Su et al., 2020) showed that *Tetranychus urticae* feeding significantly suppressed rutin and quercetin levels in tomato plants, increasing the plant’s attractiveness to *B. tabaci*. However, the exogenous application of 20 μL of 10 μM rutin or quercetin dissolved in 1% ethanol resulted in concentrations similar to those of non-infested plants, without causing detrimental effects on total leaf flavonoids, as confirmed by electrical penetration graph recordings. Treated plants exhibited shorter durations of probing, watery salivation, and phloem ingestion (**Figure 2**). The impact of rutin on enhancing tomato plant resistance has also been demonstrated through seed treatment. Tang et al. (2023) treated tomato seeds with varying concentrations of rutin (1, 2, 5, 10, and 20 mM) to assess impacts on seedling vigor, growth, whitefly feeding behavior, and *B. tabaci* performance. Apart from a negative impact on seedling root growth in samples treated with 10 mM and 20 mM of rutin, treatment with flavonoid did not compromise seed germination or plant growth. Furthermore, rutin enhanced plant resistance and reduced adult fecundity and nymph development rate. Similarly, Zhang et al. (2025) reported that rutin treatment of tomato seeds did not compromise plant growth and development, while inducing a primed defensive state. Seed priming resulted in long-lasting, broad-spectrum resistance to herbivores, including *B. tabaci*. Mechanistically, treatment with rutin enhanced the accumulation of jasmonic acid and jasmonic acid-isoleucine in seeds, consequently priming tomato anti-herbivore defenses that depend on the jasmonic acid signaling pathway.

Yao et al. (2019) showed similar findings using near-isogenic lines (NIL-PH) of tomato differing in flavonoid content. In settlement preference assays, the flavonoid-rich NIL-PH line was less attractive to whiteflies than the flavonoid under-producing near-isogenic line (NIL-GH), signaling the deterrent effect of these SMs. Furthermore, resistance in NIL-PH lines limited TYLCV spread, highlighting their importance in both herbivore and vector control. The high resistance observed in high-flavonoid-producing near-isogenic lines (NILs) may be due to these SMs inhibiting whitefly effector proteins. A study by Su et al. (2025) with two NILs demonstrated that cultivated tomatoes with high flavonoid levels suppress the expression of *B. tabaci* effector protein 3 (*BtE3*), a salicylic acid elicitor that influences the host plant’s susceptibility by downregulating genes in the jasmonic acid-dependent defense pathway. Diet feeding assays and CRISPR/Cas9-generated *S. lycopersicum flavonol synthase* mutants showed that quercetin and rutin could inhibit BtE3 gene expression, connecting resistance in NIL-PH tomatoes to these compounds. Similarly, Li et al. (2025) demonstrated through a study with NILs with distinct flavonoid levels that this SM class may confer resistance to *B. tabaci* through constitutive and induced plant resistance mechanisms. Electrical penetration graph analysis showed reduced probing time and phloem ingestion on the NIL-PH compared to the variety with low flavonoid content. The authors stated that whitefly infestation on NIL-PL induced stronger reactive oxygen species accumulation through NADPH oxidase, while simultaneously upregulating callose synthase gene expression and inducing callose deposition in the sieve elements. As a result, whitefly phloem uptake and performance were substantially reduced in the NILs rich in flavonoids. Although the authors state that further research is necessary to fully understand the mechanism underlying the interaction between whiteflies and tomato lines, their findings provide a promising direction for breeding insect-resistant tomato varieties.

At the molecular level, a study by Xia et al. (2021) revealed a plant-specific and horizontally transferred gene in *B. tabaci*, *BtPMaT1*, that encodes a phenolic glucoside malonyltransferase involved in detoxifying plant phenolic glycosides. Metabolite profiling using Ultra-High Performance Liquid Chromatography coupled with Quadrupole Time-of-Flight Mass Spectrometry (UPLC-QTOF/MS) showed increased mortality in adult whiteflies exposed to compounds such as kaempferol 3-O-glucoside, kaempferol 7-O-glucoside, phenyl β-D-glucoside, phlorizin, and rhaponticin. Silencing *BtPMaT1* enhanced the toxicity of these glycosides to whiteflies, confirming its function in mitigating phenolic-based defenses in host plants.

The antixenosis effect triggered by phenolic compounds is not limited to insect pests and may also influence beneficial organisms in the environment. For instance, Yang et al. (2023b) investigated the potential non-target effects of tomato phytochemicals on the beneficial predator *Orius sauteri*. Comparisons between *O. sauteri* confined with NIL with distinct flavonoid levels demonstrated that the oviposition, nymphal survival rate, and development of the predator decreased significantly in resistant plants, even when provided with prey. Preference assays also showed how *O. sauteri* females preferred NILs with low flavonoid levels for oviposition. Their results reveal an unintended negative impact of host-plant resistance strategies on beneficial organisms, signaling broader ecological ramifications linked to a phytochemical-based defense.

### 5.3 Nitrogen-containing SMs

Nitrogen-containing SMs are defined by the presence of nitrogen atoms in their molecular structures and are categorized into alkaloids and cyanogenic glycosides (War et al., 2020; Yactayo-Chang et al., 2020). Cyanogenic glycosides consist of an α-hydroxynitrile moiety linked to a sugar residue derived from amino acids (Beran et al., 2019). In contrast, the definition of alkaloids is complex, as the boundaries between these compounds and others are unclear (Kabera et al., 2014). The term “alkaloid” generally refers to nitrogen-containing organic compounds, excluding proteins, peptides, and nucleic acids (Zhan et al., 2022).

Alkaloids are divided into three groups based on their chemical structure and the biosynthetic origin of their nitrogen atom (Abegaz and Kinfe, 2019; Lichman, 2021). In true alkaloids and proto-alkaloids, nitrogen originates from amino acids such as ornithine, lysine, tyrosine, anthranilic acid, picolinic acid, histidine, and tryptophan (Abegaz and Kinfe, 2019). However, in proto-alkaloids, the nitrogen is not incorporated into a heterocyclic ring (Lichman, 2021). In pseudoalkaloids, nitrogen is introduced to a molecule through transaminase reactions, rather than directly from an amino acid precursor (Divekar et al., 2022).

Tomatoes, particularly when unripe, are abundant in steroidal alkaloids, a type of pseudoalkaloid, and their glycosylated derivatives (Fiesel et al., 2022; Wang et al., 2024a). These molecules are composed of an aglycone structure, which includes the nitrogen atom, and a carbohydrate moiety linked to the 3-OH position (Cárdenas et al., 2015). One of the most significant steroidal glycoalkaloids of *S. lycopersicum* is tomatine, a steroidal saponin comprised of a tomatidine molecule (the aglycone) attached to the tetrasaccharide group (Dall’Asta et al., 2009).

The influence of plant alkaloids over insects is quite diversified, including neurotoxicity, reduced fecundity, mutagenicity, and cellular and physiological process disturbance, which leads to sublethal damage to various tissues and organs (Chowański et al., 2016; Züst and Agrawal, 2016). Glycoalkaloids have been shown to affect the endocrine system of insects, disturbing critical biological processes such as molting and metamorphosis (Oberdörster et al., 2001).

Despite their known bioactivity, studies investigating the relationship between alkaloid content and resistance to *B. tabaci* in tomato are limited. One of the few relevant studies, conducted by Van Gelder and De Ponti (1987), measured α-tomatine and other steroidal glycoalkaloids (SGAs) in the fruits of whitefly-resistant tomato lines. However, the focus was limited to assessing the safety of these lines for human consumption, and the potential impacts of the compounds on whiteflies were not investigated.

Nonetheless, several studies described the allelochemical properties of *Solanum* alkaloids against several other insect pests. For instance, bioactivity has been reported against *Drosophila Melanogaster* wild-type flies (Ventrella et al., 2016), *Tribolium castaneum* Herbst (Nenaah, 2011), *Sitophilus oryzae* L (Nenaah, 2011), and *Frankliniella occidentalis* (Bac-Molenaar et al., 2019). Furthermore, recent omics and genetic research provide indirect evidence of the role of alkaloids in tomato defense. However, research on validation for *B. tabaci* remains limited. In *Solanaceae*, genomic and biochemical studies have characterized the diversification of steroidal glycoalkaloid biosynthetic pathways driven by clustered glycoalkaloid metabolism (GAME) genes and enzymes such as UDP-glycosyltransferases and cytochrome P450s (Cárdenas et al., 2015; Lichman, 2021). Research shows that several transcription and light-responsive factors regulate SGA biosynthesis (Nakayasu et al., 2018; Sinha et al., 2024). The jasmonate-responsive Ethylene Response Factor (ERF) transcription factor JRE4 is essential for the coordinated transcription of multiple SGA biosynthetic genes. A mutation in JRE4 that disrupts DNA binding led to a significantly lower accumulation of the SMs, which was linked with decreased resistance to *Spodoptera litura* caterpillar feeding (Nakayasu et al., 2018). Similarly, ELONGATED HYPOCOTYL 5 homolog, SIHY5, regulates SGA biosynthesis as a key light-responsive factor that influences light-mediated synthesis by interacting with the promoter regions of essential biosynthetic genes. Regulation by SIHY5 resulted in increased SGA accumulation and greater resistance to fungal infection (Sinha et al., 2024).

Bai et al. (2024) characterized a tomato T-DNA insertional mutant and discovered a GAME enhancer 1 (GE1) linked to the GAME cluster gene regions on chromosome 7. The distal enhancer GE1 recruits the MYC2-GAME9 transcriptional complex to control the expression of GAME cluster genes. During domestication, breeding programs favored selecting GE1 to lower toxic SGA levels. Evolutionary research by Jozwiak et al. (2025) on alkaloid stereochemistry revealed how GAME8 cytochrome P450 hydroxylases control the stereochemistry of the C25 chiral center, directly affecting the SGA structure and biological activity, consequently influencing the role of the SM in plant defense. Collectively, these findings suggest that *Solanum* alkaloids may hold untapped potential as defense compounds against *B.tabaci*, warranting further investigation into their roles in tomato resistance (**Supplementary Table S2**).

### 5.4 Acylsugars

Acylsugars are specialized protective metabolites widely distributed within the *Solanaceae* plant family and are frequently linked to arthropod resistance in the *Solanum* genus (Rodríguez-López et al., 2012; Mutschler et al., 2023; Smeda et al., 2023). These allelochemicals consist of a sugar core, such as glucose, sucrose, and, less commonly, inositol, esterified with multiple fatty acid (acyl) chains (Fan et al., 2019; Fiesel et al., 2022; Moghe et al., 2023). Despite their simple building blocks, acylsugars exhibit remarkable structural diversity due to several variations in sugar core, acyl chain length, branching patterns, and substitution positions (Fan et al., 2019; Vendemiatti et al., 2024).

To better understand this structural variability, *S. lycopersicum* has been used as a model species for elucidating the acylsugar biosynthetic pathway (Fiesel et al., 2022; Vendemiatti et al., 2024) Predominant chemotypes across *Solanum* accessions from distinct locations reflect genetic drift in the biosynthetic pathway, suggesting local adaptation of acylsugar yield and composition to varying edaphoclimatic conditions (Leckie et al., 2016).

Acylsugars are among the most prominent allelochemicals of tomato linked to insect resistance (Rodríguez-López et al., 2012; Leckie et al., 2016). These compounds can act as a direct defense by immobilizing and causing anoxia in insects or as an indirect defense by affecting tritrophic interactions, thus reducing pest success (**Figure 2**, **Supplementary Table S2**) (Weinhold and Baldwin, 2011; Fan et al., 2019; Moghe et al., 2023). In a study comparing resistant line ABL 14–8 with susceptible cultivar Rodríguez-López et al. (2011) found that *B. tabaci* eluded settling and feeding on ABL 14–8 due to the high density of type IV glandular trichomes and high levels of acylsucroses in its leaflets. A follow-up study revealed that whiteflies preferred to settle on the abaxial side of Moneymaker leaves. In contrast, no side preference was observed in ABL 14-8, suggesting that trichome exudates on the abaxial side altered whitefly behavior (Rodríguez-López et al., 2012).

A similar correlation between acylsugar content and host resistance was also reported by Narita et al. (2023). In their study, genotype LA716 exhibited high whitefly mortality, reduced inoculation efficiency of severe rugose virus in tomatoes, and limited stylet penetration. These positive results were attributed to the high abundance of type IV glandular trichomes producing acylsugars on the abaxial side of the leaf.

Host resistance has also been successfully bred into progenies of tomato cultivars with suitable traits (Silva et al., 2014; Dias et al., 2016). F_3_ tomato families, derived from an interspecific cross between the susceptible parental line LAM-148 and the highly resistant parental line TO-937-15, exhibited varying glandular trichome density and acylsugar content. This variation enabled classification of progenies into susceptibility groups, with higher glandular trichome density and acylsugar content correlating with lower whitefly oviposition (Silva et al., 2014). Dias et al. (2016) reported similar findings using genotypes derived from *S. lycopersicum* Redenção and *Solanum pennelli.* Progenies with low acylsugar content supported higher whitefly oviposition and nymph densities, while those with higher acylsugar levels deterred insect colonization and altered host preference.

Field studies have confirmed the potential of acylsugar-mediated resistance. Pandey et al. (2023) explored experimental tomato lines derived from the wild relative *S. pennellii* LA716 and found reduced whitefly egg, nymph, and adult populations, as well as enhanced establishment of the predatory mite *Amblyseius swirskii* Athias-Henriot compared to commercial cultivars. Interestingly, whitefly deterrence was more pronounced under greenhouse conditions, suggesting context-dependent efficacy. Similarly, Smeda et al. (2023) showed that acylsugars introgressions into field-grown tomato lines significantly reduced egg and nymph populations and suppressed TYLCV incidence for 51 days.

While the significance of acylsugars in *B. tabaci* is well-established, their complexity presents challenges. The observed variability in insect response could be influenced by the sugar backbone, fatty acid (acyl) chain profile, concentration, or interactions among these factors (Mutschler et al., 2023). A study by Leckie et al. (2016) on the influence of acylsugar components on arthropod oviposition demonstrated that all treatments suppressed whitefly oviposition, but efficacy varied. Treatment with acylsugars extracted from CU071026, rich in acylsucroses and i-C5 fatty acid chains, exhibited the lowest efficacy among acylsugar treatments. Intermediate efficacy was achieved with acylsugars isolated from LA716 and LA1732 lines, characterized by acylglucoses and high levels of i-C4 fatty acid chains. The highest suppression was achieved with extracts from LA2560 and LA1376, which contained both glucose and sucrose cores and high levels of i-C5 chains.

Recent advances have also improved the understanding of acylsugar composition and its role in resistance. Kortbeek et al. (2021) applied random forest modeling to identify specific SMs from several tomato genotypes that conferred resistance to *B. tabaci* and *Frankliniella occidentalis*. Their analysis indicated that resistance was more closely associated with chemical composition than with trichome density. Specifically, Type VI glandular trichome density did not correlate with survival rates of insect pests, whereas Type I and IV glandular trichomes showed negative correlations. The model also predicted two key metabolites, S3:15 and S3:21, as candidates responsible for reducing *B. tabaci* survival (Kortbeek et al., 2021).

## 6 Future directions

Decades of selective breeding aimed at improving tomato yield and fruit quality have resulted in the loss of essential host resistance traits. Restoring these traits through the introgression of genes from wild tomatoes remains a promising and environmentally sustainable approach to enhance resistance to *B. tabaci*. Although managing whiteflies in tomato crops is difficult, increasing evidence supports the effectiveness of leveraging endogenous SMs for pest suppression. However, the available literature should be interpreted with caution, as most studies are conducted under controlled environments. Under open-field conditions, factors such as temperature, humidity, soil fertility, crop developmental stage, and the *B. tabaci* cryptic species can affect the success of an SM-based approach. Field assays with acylsugar introgressions showed suppression of TYLCV spread and *B. tabaci* oviposition and development (Smeda et al., 2023). However, the variability in the data highlights the need for additional research under different environmental conditions to determine how the environment influences *B. tabaci* preference accurately. Similarly, Pandey et al. (2023) reported a greater resistance to whiteflies in experimental tomato lines grown in greenhouse conditions compared to those in open-field conditions, underscoring the importance of evaluating context-dependent efficiency.

Given the high adaptability of *B. tabaci*, sustainable pest control will require a multilayered and dynamic approach. The insect can circumvent phenolic-based plant defenses by detoxifying phenolic glycosides (Xia et al., 2021). *B. tabaci* hijacked a plant−derived phenolic glucoside malonyltransferase (BtPMaT1) by horizontal gene transfer, which enables the neutralization of toxic phenolic SMs. Additionally, recent studies have identified tomato flavonoids that suppress salivary effectors that downregulate host plant defenses (Su et al., 2025), signaling the constant adaptation of tomatoes and whiteflies to control the plant-insect relationship. Therefore, the high adaptability of *B. tabaci* to chemical control and the available literature on adaptability to specific SMs signals a similar behavior if resistance relies on a single metabolite class.

In addition to investigating the impacts of SMs on *B. tabaci*, it is essential to assess the non-target effects of an SM-based control approach on the environment. Flavonoid-rich NILs negatively affected *Orius sauteri* performance (Yang et al., 2023b), whereas tomato lines rich with acylsugar hindered the growth and foraging of *Amblyseius swirskii* (Pandey et al., 2023), which underscores the importance of assessing the compatibility of SM-based approaches with other techniques used in IPM.

Emerging biotechnologies enable precise regulation of SM pathways while reducing trade-offs with yield, fruit quality, and palatability. Metabolite-assisted selection helps breeders monitor and improve SM profiles, while gene-editing tools like CRISPR offer opportunities to fine-tune biosynthetic pathways or regulation factors, such as GAME enhancers, to boost defense without sacrificing produce quality (Huang et al., 2022; Bai et al., 2024).

Future research should focus on studies in open-field conditions. Although data on *B. tabaci* resistance is primarily based on greenhouse or laboratory settings, real-world environments introduce additional variables that can influence pest–plant interactions and treatment outcomes. Testing these strategies under diverse environmental conditions will be crucial for translating laboratory successes to the field. Additionally, IPM strategies should aim to integrate existing technologies. Endogenous resistance in tomato varieties could be enhanced by inducing early glandular trichome synthesis through exposure to biotic or abiotic stress. This approach could also be paired with intercropping systems that include whitefly-resistant plants like marigolds. Exploring the combined effects of induced resistance and companion planting offers a promising direction for more effective whitefly control. Similarly, several studies have focused on evaluating the effects of a single class of SMs, but research on the synergistic or antagonistic interactions between different SM classes is limited. Investigating the overlapping effects of terpenes with acylsugars or phenolic compounds could provide an intriguing approach for managing *B. tabaci* pest populations.
