# Supplementary material for: Tomato secondary metabolites as natural regulators of Bemisia tabaci behavior and performance: current applicability and prospects
Source: Front Plant Sci. 2026 Jan 15;16:1704832. doi: 10.3389/fpls.2025.1704832 (PMC12851975; doi:10.3389/fpls.2025.1704832)
Supplement: Supplementary file 1 [file Supplementaryfile1.docx]

**SUPPLEMENTARY MATERIAL – Frontiers in Plant Science**

**Tomato secondary metabolites as natural regulators of *Bemisia tabaci* behavior and performance: Current applicability and prospects**

Victor Hugo Maldonado Machado da Cruz ^a^^,b^, Thiago Rutz da Silva ^a^, Paulo Gimenez Cremonez ^c^, Andre Luiz Biscaia Ribeiro da Silva ^a^, Jesui Vergilio Visentainer^b^, Camila Rodrigues ^a*^

*^a^ Department of Horticulture, Auburn University, Auburn, AL 36849, United States*

*^b^ Department of Chemistry, State University of Maringá, Maringá, Paraná, 87020-900, Brazil*

*^c^ Department of Entomology and Plant Pathology, Auburn University, Auburn, AL 36849, United States*

*Corresponding author e-mail: crodrigues@auburn.edu

**Table S1:** Summary of studies identifying secondary metabolites in tomato associated with resistance to *Bemisia tabaci*.

| **Components** | **Secondary metabolite class** | **Experimental assays** | **Major results** | **Reference** |
| --- | --- | --- | --- | --- |
| Zingiberene | Sesquiterpene | Free-choice bioassays with wild tomatoes and electroantennography | Zingiberene, curcumene, *p*-cymene, α-terpinene, and α-phellandrene exhibited repellent properties, decreasing host attractiveness | (Bleeker et al., 2009) |
| Curcumene | Sesquiterpene |  |  |  |
| *p*-cymene | Monoterpene |  |  |  |
| α-terpinene | Monoterpene |  |  |  |
| α-phellandrene | Monoterpene |  |  |  |
| β-myrcene | Monoterpene |  |  |  |
| γ-terpinene | Monoterpene |  |  |  |
| 7-Epizingiberene | Sesquiterpene | No-choice and free-choice bioassays and electroantennography | 7-Epizingiberene and R-curcumene act as semiochemicals and modify the host preference of *B. tabaci.* | (Bleeker et al., 2011) |
| R-curcumene | Sesquiterpene |  |  |  |
| S-curcumene | Sesquiterpene |  |  |  |
| Zingiberene | Sesquiterpene |  |  |  |
| 7-epi-9-hydroxy-zingiberene | Sesquiterpene | No-choice bioassays and antibacterial assays | 7-epi-9-hydroxy-10,11-epoxy-zingiberene caused a concentration-dependent reduction in whitefly population. Both compounds exhibited antimicrobial activity | (Zabel et al., 2021) |
| 7-epi-9-hydroxy-10,11-epoxy-zingiberene | Sesquiterpene |  |  |  |
| Limonene | Monoterpene | Glasshouse experiments with French marigolds as a preventive or emergency measure against whitefly infestations | Intercropping proved effective when implemented as a preventive measure, but not as an emergency measure. Limonene dispensers decreased the pest load by reducing plant attractiveness | (Conboy et al., 2019) |
| Limonene | Monoterpene | Commercial glasshouse setting with dispensers of limonene, methyl salicylate, or a combination of both | Treatment with limonene dispensers provided the best results among the three treatments investigated | (Conboy et al., 2020) |
| Caryophyllene | Sesquiterpene | Two-leaf, six-leaf, and ten-leaf of ‘Micro Tom’ tomato treated with different levels of caryophyllene | Treatment with 25 µmol L^-1^ demonstrated the highest repellent effect. The treatment also enhanced the synthesis of several endogenous terpenoids | (Xu et al., 2024) |
| Phenol | Simple Phenol | Twenty tomato genotypes were evaluated under field conditions at different seasons | Higher levels of phenol and tannin were variables linked to resistance to *B. tabaci* in resistant genotypes | (Anu et al., 2021) |
| Tannin | Polyphenolic compound |  |  |  |
| Phenol | Simple Phenol | Screening of Indian tomato genotypes for resistance to *B. tabaci* and TYLCV | Resistant genotypes exhibited high levels of total phenol content | (Pal et al., 2021a) |
| α-terpinene | Monoterpene | Host preference was assessed through dual-choice assays in greenhouses. | *B. tabaci* herbivory decreased the contents of rutin, kaempferol rhamnoside, quercetin trisaccharide, and 3-O-methyl myricetin, and increased the attractiveness of tomatoes to conspecific feeders, signaling their impact of host preference assays | (Su et al., 2018) |
| α-phellandrene | Monoterpene |  |  |  |
| Rutin | Flavonoid glycoside |  |  |  |
| Kaempferol rhamnoside | Flavonoid glycoside |  |  |  |
| Quercetin trisaccharide | Flavonoid glycoside |  |  |  |
| 3-O-methyl myricetin | Flavonoid glycoside |  |  |  |
| Rutin | Flavonoid glycoside | A food web of *Solanum lycopersicum, B. tabaci, T. urticae,* and TYLCV was used to evaluate TYLCV transmission | Low levels of rutin and quercetin trisaccharide increased susceptibility to *B. tabaci*. Treatment with flavonoids decreased settling, probing, and feeding | (Su et al., 2020) |
| Quercetin trisaccharide | Flavonoid glycoside |  |  |  |
| Rutin | Flavonoid glycoside | Treatment of *Solanum lycopersicum* with different rutin concentrations | Treatment with rutin on concentrations below 10 mM enhanced resistance to *B. tabaci* without affecting growth | (Tang et al., 2023) |
| Flavonoids | Flavonoids | No-choice and dual-choice assessments between near-isogenic lines | Near-isogenic lines with high levels of flavonoids deterred landing, settling, probing, phloem-feeding, and TYLCV transmission | (Yao et al., 2019) |
| Kaempferol 3-O-glucoside | Phenolic glucosides | Bioinformatic, molecular, and biochemical approaches to identify plant-derived phenolic glucoside malonyltransferase gene *BtPMaT1*, and insect performance assays to verify its effect on tomato-whitefly interactions | The increase in adult whitefly mortality after silencing *BtPMaT1* demonstrates the toxic effects of the five SMs | (Xia et al., 2021) |
| Kaempferol 7-O-glucoside | Phenolic glucosides |  |  |  |
| Phenyl β-D-glucoside | Phenolic glucosides |  |  |  |
| Phlorizin | Phenolic glucosides |  |  |  |
| Rhaponticin | Phenolic glucosides |  |  |  |
| Acylsucroses | Acylsugar | No-choice and free-choice condition assays to compare behavior towards tomato lines ABL 14-8 and Moneymaker | The higher concentration of type IV glandular trichomes and acylsucroses in ABL 14-8 hindered infestation and TYLCV spread | (Rodríguez-López et al., 2011) |
| Acylsucroses | Acylsugar | No-choice and free-choice settlement preference assessment using ABL 14-8 and Moneymaker | No preference for the abaxial or adaxial side of leaves was observed in the ABL 14-8 tomato line. The change in *B. tabaci* behavior was attributed to a higher content of acylsucrose exudates | (Rodríguez-López et al., 2012) |
| Not specified | Acylsugar | Assessment of the survivorship of *Tomato severe rugose virus* (ToSRV) and probing behavior of *B. tabaci* on three tomato genotypes (Santa Clara, LA716, and Ivety | Genotype LA716 caused a high mortality of adult whiteflies (>95%), and limited ToSRV inoculation by reducing probing-associated processes | (Narita et al., 2023) |
| Not specified | Acylsugar | Free-choice and no-choice assays for oviposition and settling | Resistance to *B. tabaci* was linked to a high concentration of acylsugar-producing type IV glandular trichomes | (Silva et al., 2014) |
| Not specified | Acylsugar | Randomized block design with 12 treatments for F_2_ genotypes and 14 treatments for F_2_RC_1_ genotypes. Genotypes were evaluated based on oviposition and nymph population on leaflets | Tomato genotypes with high acylsugar levels, similar to *Solanum pennelli,* modified host preference, decreasing host attractiveness | (Dias et al., 2016) |
| Not specified | Acylsugar | Two greenhouse experiments to evaluate the role of tomato lines and *A. swirskii* application in whitefly population suppression and the non-pest food effects on *A. swirskii* establishment and whitefly suppression | Smaller populations of eggs, nymphs, and adults were detected in tomato lines derived from the wild relative *Solanum pennellii* LA716 | (Pandey et al., 2023) |
| Acylsucrose | Acylsugar | Field trials in the spring and fall of 2014, and spring of 2015 to evaluate the effect of tomato entries, acylsugar composition, and content on whitefly oviposition, survival, development, and TYLCV incidence | Acylsugar amount was linked to smaller egg and nymph populations, and to the incidence of TYLCV. Composition of acylsugars demonstrated an effect on its efficiency | (Smeda et al., 2023) |
| Acylglucose | Acylsugar |  |  |  |
| Acylsucrose | Acylsugar | Evaluation of the effects of multiple rates of acylsugar extracts from *S. pennellii* accessions and breeding line CU71026 on oviposition in non-choice and choice bioassays | Positive outcomes were achieved with all treatments, but the highest yield was achieved with a mix of acylsucroses and acylglucoses, rich in i-C5 fatty acids | (Leckie et al., 2016) |
| Acylglucose | Acylsugar |  |  |  |
| S3:15 | Acylsugar | Random forest analyses to identify SMs and pinpoint chemicals that confer resistance to *Bemisia tabaci* and *Frankliniella occidentalis* | Resistance to whitefly is linked to the composition of the exudate rather than the density of glandular trichomes. S3:15 and S3:21 was associated resistance to *B. tabaci* | (Kortbeek et al., 2021) |
| S3:21 | Acylsugar |  |  |  |

**Table S2:** Comparative table of the effect of tomato secondary metabolites on *Bemisia tabaci.*

| **Secondary metabolites class** | **Compound** | **Mode of action** | **Outcomes and recommendations** | **Limitations** | **References** |
| --- | --- | --- | --- | --- | --- |
| Terpenes | Zingiberene, 7-epi-zingiberene, curcumene, p-cymene, α-terpinene, limonene, caryophyllene | Manipulates odor cues, altering host preference;  Attraction of natural predators of *B.tabaci.* | Positive outcomes observed in laboratory and greenhouse settings. Further research is needed for open-field application. An open-field application could potentially be used preventively. | Volatility;  Dispersion in an open-field setting;  Timing. | (Bleeker et al., 2009, 2011; Conboy et al., 2019, 2020; Ayelo et al., 2021; Xu et al., 2024) |
| Phenolic compounds | Rutin, quercetin, kaempferol glycosides; tannins; phenolic glucosides | Modulated probing and feeding times;  Induces oxidative stress. | Strong inhibition of *B. tabaci* performance under greenhouse and laboratory settings. Further research is needed for open-field application. | Reported cases of whiteflies detoxifying toxic phenolic compounds through gene adaptation;  Undesirable effect on non-target insects;  High doses can negatively impact plant quality parameters | (Su et al., 2018, 2020; Yao et al., 2019; Anu et al., 2021; Pal et al., 2021b; Xia et al., 2021; Tang et al., 2023; Yang et al., 2023b, 2023a) |
| Nitrogen-containing SMs | Steroidal glycoalkaloids (SGAs) | Potential antibiosis | Literature on the effects of SGAs on *B. tabaci* is very limited. | Potential fruit palatability trade-off;  Safety issues, for instance, α-tomatine is toxic to humans at high concentrations;  Limited evidence on this class of SMs | (Cárdenas et al., 2015; Nakayasu et al., 2018; Lichman, 2021; Bai et al., 2024; Sinha et al., 2024; Jozwiak et al., 2025) |
| Acylsugars | Acylglucoses and Acylsucroses | Settling deterrence;  Immobilization and anoxia;  Limits stylet penetration | Effective against whiteflies under greenhouse and laboratory settings;  Acylsugar structure significantly influences efficiency. | Compatibility issues with natural predators of *B. tabaci;*  Environmental variability | (Rodríguez-López et al., 2011, 2012; Silva et al., 2014; Leckie et al., 2016; Kortbeek et al., 2021; Narita et al., 2023; Pandey et al., 2023; Smeda et al., 2023) |
